# EXPLORING CENTRAL SYMPTOMS OF SUBTHRESHOLD DEPRESSION IN OLDER ADULTS: A NETWORK ANALYSIS

**DOI:** 10.1093/geroni/igae098.3431

**Published:** 2024-12-31

**Authors:** Rendong He, Li Chen, Yiming Qiu, Junxin Li

**Affiliations:** Jilin University School of Nursing, Changchun, Jilin, China; Jilin University, Changchun, Jilin, China (People’s Republic); Jilin University, Changchun, Jilin, China (People’s Republic); Johns Hopkins School of Nursing, Baltimore, Maryland, United States

## Abstract

Subthreshold depression (StD), a precursor to major depressive disorder, has not been well studied in older adults. Identifying central symptoms among various symptoms within the mental disorders enhances understanding of mental disorder structures and informs prevention strategies. We investigated the central symptoms of StD in community-dwelling older adults in China in a cross-sectional study. Depressive symptoms were assessed by prime care physicians during older adults’ well visits using the Hamilton Depression Scale-17 (HAMD-17). Those with StD (7≤ HAMD-17< 17) were included in the study (n = 424). We also collected data on demographics, depressive symptoms, anxiety symptoms, and cognitive function. The network model was constructed using the “least absolute shrinkage and selection operator” technique with the tuning parameters minimizing the extended Bayesian information criterion. Node centrality was assessed using Expected Influence (EI). Network stability was examined with a case-dropping subset bootstrap featuring a correlation stability coefficient (CS). The sample consisted of 53.5% females who, on average, were 69.69 ± 6.80 years and with a HAMD-17 score of 10.46 ± 3.21. Among the 17 symptoms, the two most central symptoms were “Loss of interest in work or activities” (EI = 0.51) and “Depressed Mood” (EI = 0.41). The correlation between these two symptoms is the strongest (Edge weight = 0.26) among all symptoms. The estimated network has a good stability (CS = 0.44). Our findings suggest that “Loss of interest in work or activities” and “Depressed Mood” are potential intervention targets for StD in older adults.

